# Cognitive Trajectories After Postoperative Delirium: Lessons Learned After 6 Years of Follow-Up in the SAGES Cohort

**DOI:** 10.1093/geroni/igaf122.1054

**Published:** 2025-12-31

**Authors:** Zachary Kunicki, Tammy Hshieh, Long Ngo, Douglas Tommet, Tamara Fong, Thomas Travison, Richard Jones, Sharon Inouye

**Affiliations:** Warren Alpert Medical of Brown University, Providence, Rhode Island, United States; Brigham and Women’s Hospital, Boston, Massachusetts, United States; Beth Israel Deaconess Medical Center, Boston, Massachusetts, United States; Brown University, Providence, Rhode Island, United States; Beth Israel Deaconess Medical Center, Boston, Massachusetts, United States; Brown University, Providence, Rhode Island, United States; Hebrew SeniorLife & Harvard Medical School, Boston, Massachusetts, United States

## Abstract

The Successful Aging after Elective Surgery (SAGES) cohort is the longest-running prospective study of cognitive trajectories, examining how delirium can influence long-term cognitive outcomes. Two major papers from the SAGES research group — Inouye et al. (2016) and Kunicki et al. (2023) — demonstrate that delirium is associated with accelerated cognitive aging. Using a measure of general cognitive performance, derived using latent variable modeling of 11 cognitive tests spanning domains of attention, memory, language, and executive functioning, Inouye et al. (2016) illustrates a multifaceted post-surgical trajectory which is characterized by a decline one month after surgery, recovery to above the preoperative baseline at two months, followed by a gradual decline. Kunicki et al. (2023) further highlights the complexity of this trajectory by showing there is an additional period of little change until 36 months, but overall that the rate of cognitive decline in patients who experienced delirium is 40% faster than in those who did not. This symposium will review the key findings from these papers, emphasizing how delirium has a lasting and significant impact on cognitive trajectories. Lessons learned from these analyses, along with potential directions for future research, will also be discussed.

